# The Role of Urgent Care Clinics in Alleviating Emergency Department Congestion: A Systematic Review of Patient Outcomes and Resource Utilization

**DOI:** 10.7759/cureus.81919

**Published:** 2025-04-08

**Authors:** Nasser Abullah N Alaqil, Bader Ghanem Alanazi, Saleh Abdullah Ali Alghamdi, Mohammed Ghanem Alanazi, Ahmed Abdulrahman Abdullah Alghamdi, Ahmed Mayudh Oudah Almalki, Faisal Baalqasim Hassan Alamri

**Affiliations:** 1 Family Medicine, Urgent Care Unit, Al Kharj Armed Forces Hospital, Al Kharj, SAU; 2 Family Medicine, Prince Sultan Military Medical City, Riyadh, SAU; 3 Family Medicine, Armed Forces Hospital Southern Region, Khamis Mushait, SAU; 4 Emergency Medicine, Riyadh Armed Forces Hospital, Riyadh, SAU; 5 Emergency Medicine, Armed Forces Hospital, Jubail, SAU; 6 Emergency Medicine, Northern Area Armed Forces Hospital, King Khalid Military City, Khamis Mushait, SAU; 7 Preventive Medicine, King Fahd Armed Forces Hospital, Jeddah, SAU

**Keywords:** emergency department, er congestion, healthcare efficiency, patient outcomes, urgent care clinics, wait times

## Abstract

This systematic review evaluates the impact of standalone urgent care clinics (UCCs) on reducing emergency department (ED) congestion by assessing their influence on patient volumes, wait times, and length of stay. Additionally, it examines UCCs' role in improving patient satisfaction, optimizing healthcare resource utilization, and enhancing cost-effectiveness. A comprehensive literature search was conducted across five major databases (PubMed, ScienceDirect, Google Scholar, Semantic Scholar, and Directory of Open Access Journals {DOAJ}) using Medical Subject Headings (MeSH) and relevant keywords. Twelve peer-reviewed studies published between 2015 and 2024 met the inclusion criteria, focusing on the relationship between UCC implementation and ED performance metrics. The findings indicate that UCCs significantly reduce ED visits, particularly for non-urgent cases, leading to shorter wait times and improved resource allocation. UCCs also enhance healthcare accessibility for underserved populations and are associated with higher patient satisfaction. Cost-effectiveness analyses suggest that UCCs lower overall healthcare expenditures by reducing unnecessary ED visits. However, challenges such as workforce redistribution and regional disparities in UCC effectiveness remain. Integrating UCCs into healthcare systems reduces ED congestion, improves operational efficiency, lowers costs, and enhances patient satisfaction. Future research should explore long-term patient outcomes and strategies for better integration of UCCs within broader healthcare networks.

## Introduction and background

Emergency departments (EDs) are the cornerstone of healthcare systems worldwide, providing life-saving care for critically ill and injured patients. However, EDs are increasingly overwhelmed by overcrowding, prolonged wait times, high operational costs, and resource constraints, which compromise their ability to deliver timely and effective care. This persistent issue can lead to delayed diagnoses, decreased patient satisfaction, and adverse clinical outcomes. Alarmingly, research estimates that up to 40% of ED visits are for non-urgent conditions, which could have been managed in alternative care settings [1-4].

The growing crisis in emergency care is driven by several factors, including increased patient demand, limited hospital capacity, and insufficient access to timely primary care services. Many individuals, particularly those without insurance or access to a regular primary care provider, turn to EDs for treatment of minor injuries, infections, or chronic conditions due to their convenience and perceived reliability. This practice places a significant burden on ED resources, diverting attention from patients with true emergencies and creating inefficiencies in healthcare delivery [5-7]. To combat these challenges, alternative care models, such as urgent care clinics (UCCs), have been introduced as viable solutions.

Urgent care clinics provide immediate, walk-in care for non-life-threatening conditions, offering patients an alternative to EDs for timely medical attention. UCCs are designed to bridge the gap between primary care and emergency services, with extended hours, no appointment requirements, and lower costs than ED visits. They cater to patients with acute but non-severe conditions, such as minor injuries, respiratory infections, and mild illnesses, without requiring ED-level intervention [8-10]. By diverting non-urgent cases away from EDs, UCCs have the potential to reduce patient volume, wait times, and operational strain on EDs, while simultaneously improving patient outcomes and satisfaction.

Studies have demonstrated a promising impact of UCCs on healthcare systems. For instance, UCCs in the United States have been associated with a 17% reduction in ED visits in certain regions, saving an estimated $3.3 billion annually by cutting non-urgent ED utilization. Historical data indicate that walk-in clinics and after-hours general practice (AHGP) services in the United Kingdom and Australia have achieved modest reductions in ER presentations, ranging from 8% to 13%, depending on the population served and healthcare system structure [11-14]. Additionally, UCCs are linked to higher patient satisfaction, as many patients appreciate the convenience of their accessible locations, shorter wait times, and lower costs compared to ED services [15,16].

Despite these benefits, the impact of UCCs is not universally consistent, and evidence regarding their effectiveness remains mixed. In some cases, UCCs have been criticized for creating workforce pressures, as healthcare staff transition from ED roles to UCCs, potentially straining resources in both settings [17]. A UK study reported that while 36% of emergency specialists believed UCCs reduced ED attendances, a significant proportion expressed skepticism about their ability to meaningfully alleviate ED workload [18]. Furthermore, some studies have shown that while UCCs reduce ED visits for low-acuity conditions, they may also contribute to increased overall healthcare spending, as the cost of widespread UCC utilization can offset savings from reduced ED visits [19,20].

Nevertheless, UCCs remain a cost-effective and patient-centred alternative for managing non-urgent conditions. The average cost of a UCC visit is estimated at $168, compared to $2,199 for freestanding ED visits, highlighting their potential to improve resource allocation and reduce healthcare expenses [21-23]. UCCs also play a critical role in improving access to care for vulnerable populations, such as Medicaid enrollees and the uninsured, who often face barriers to traditional primary care services [24]. Additionally, UCCs have shown promise in addressing healthcare disparities, particularly in underserved areas, through services tailored to specific populations, such as oncology-staffed units for cancer patients or youth-focused psychiatric care [25-27].

The rationale of this review was that it was essential to further explore the role of UCCs in addressing the challenges of ED overcrowding and optimizing healthcare delivery. This systematic review evaluated the impact of UCCs on ED operations by assessing whether their presence reduces patient volume, wait times, and length of stay in EDs. Additionally, it examined the influence of UCCs on patient outcomes, including health outcomes, satisfaction, and access to care, while also considering their cost-effectiveness and implications for healthcare resource utilization. By synthesizing the current evidence, this review aimed to provide a comprehensive understanding of how UCCs can complement traditional emergency services and alleviate the growing strain on ERs. This systematic review finds that integrating urgent care clinics into healthcare systems significantly reduces ER congestion, improves patient satisfaction, optimizes resource utilization, and contributes to overall cost-effectiveness.

## Review

Methodology

Search Strategy

A systematic and comprehensive search strategy was employed to identify relevant studies examining the impact of urgent care clinics (UCCs) on emergency department (ED) operations, specifically patient volumes, wait times, length of stay, and resource utilization, as well as patient outcomes, including satisfaction, health improvements, and cost-effectiveness.

The following five major databases were searched: PubMed, ScienceDirect, Google Scholar, Semantic Scholar, and the Directory of Open Access Journals (DOAJ). The search was conducted using a combination of keywords and Medical Subject Headings (MeSH) terms, including “Urgent Care Clinics,” “Emergency Department,” and “Patient Outcomes.”

The search began with broad terms to ensure inclusivity, such as "Urgent Care Clinics," yielding 817,453 articles. By incorporating more specific terms like "Emergency Department Urgent Care Clinic" using Boolean operators, we refined the focus to studies directly examining the relationship between UCCs and emergency departments, reducing irrelevant results and narrowing the yield to 547,701. In the next step, we focused on patient outcomes and reached 318,579 records. Then, using the search term "Impact of Urgent Care Clinic in Emergency Department and Patient Outcomes," we yielded 256,264 records.

Limiting the time frame to 2015-2024 reduced the number of records to 26,895. Applying additional filters, including restricting to English-language studies, considering only studies conducted on human populations, and selecting peer-reviewed journal articles, further reduced the included articles to 107. These articles were subsequently screened for eligibility based on relevance to the review objectives. The detailed results of the search process, including the number of articles retrieved at each stage and the application of filters, are summarized in Table 1.

**Table 1:** Relevant search results. DOAJ: Directory of Open Access Journals

Search no.	Search term	PubMed	ScienceDirect	Google Scholar	Semantic Scholar	DOAJ	Total
S1	Urgent care clinic	65,210	84,119	643,000	24,800	324	817,453
S2	Emergency department urgent care clinic	3,345	29,711	501,000	13,600	45	547,701
S3	Urgent care clinic, emergency department, and patient outcomes	821	21,476	296,000	276	6	318,579
S4	Impact of urgent care clinic on emergency department and patient outcomes	180	14,003	242,000	78	3	256,264
S5	S4 with limits: date (2015-2024)	129	8,898	17,800	65	3	26,895
S6	S5 with peer-reviewed, human journal article, and English language	17	34	45	10	1	107

Eligibility Criteria

Studies were included if they investigated the effects of UCCs on ED operations, such as changes in patient volumes, wait times, length of stay, or financial outcomes, or if they evaluated patient outcomes, such as satisfaction, health improvements, or cost-effectiveness. Articles were required to be original research studies published in peer-reviewed journals, conducted on human populations, written in English, and published between 2015 and 2024. Exclusion criteria included studies unrelated to UCCs or ED operations, non-peer-reviewed articles, conference abstracts, and studies lacking measurable outcomes relevant to the objectives of this review. Studies focusing on other healthcare models, such as telemedicine or primary care clinics, were also excluded.

Study Selection Process

The selection process followed a two-phase approach. In the first phase, titles and abstracts of the 107 articles were screened to exclude irrelevant studies. In the second phase, the full texts of potentially relevant articles were reviewed in detail to ensure they met all inclusion criteria. Discrepancies during the selection process were resolved through discussion among the reviewers, and in cases of disagreement, a third reviewer was consulted.

Quality Assessment

The quality and risk of bias in the included studies were assessed using the Newcastle-Ottawa Scale for observational studies (risk of bias in observational studies {AG1}). The Newcastle-Ottawa Scale (NOS) assesses studies based on three aspects as follows: selection (evaluating the representativeness and selection of study groups), comparability (assessing how well the study controls for confounders), and outcome/exposure (examining the quality of outcome/exposure assessment and follow-up). The maximum scores for selection, comparability, and outcome/exposure are 4, 2, and 3, respectively, resulting in a total score ranging from 0 to 9. Studies with NOS scores of 0-3, 4-6, and 7-9 were considered low, moderate, and high quality, respectively (scoring system for quality assessment {AG2}).

The NOS scores of the studies were evaluated, and those classified as low quality (score <4) were excluded to ensure that only robust and reliable evidence informed the review. The excluded studies had limitations in their design, the representativeness of their sample, the reliability of their outcome measures, or the validity of their statistical analyses. This quality assessment process strengthened the credibility of the findings and ensured their relevance to healthcare practice.

Results of the Search Strategy

The systematic search strategy yielded a total of 170 potentially relevant articles. Following the two-phase screening process, checking the reference lists, and quality assessment of articles, 10 studies were included in the final review. These studies provide valuable insights into the role of UCCs in improving ED operations, reducing congestion, and enhancing patient outcomes.

**Figure 1:**
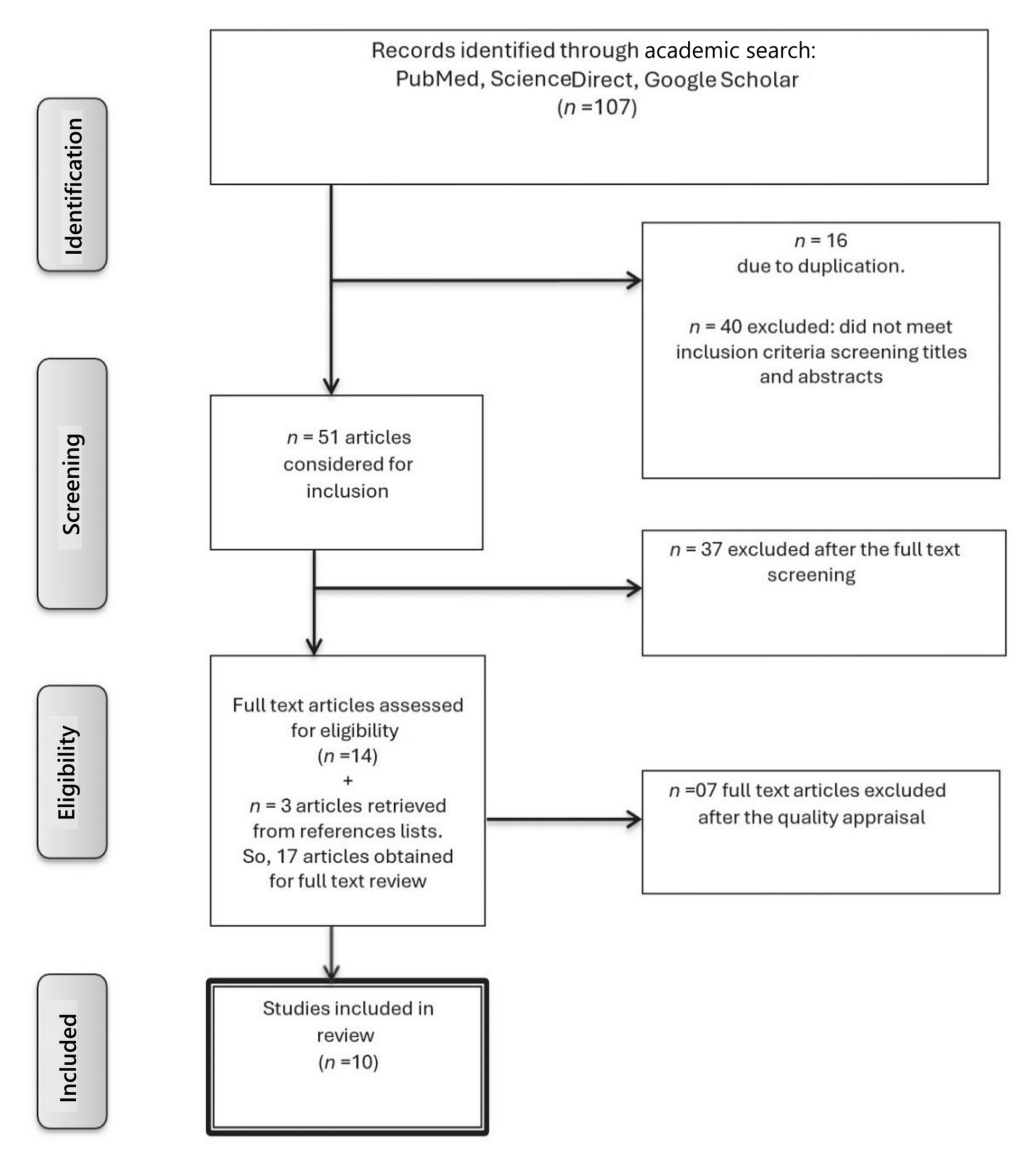
PRISMA review flow chart. PRISMA: Preferred Reporting Items for Systematic reviews and Meta-Analyses

The detailed results of the search process, including the number of articles retrieved at each stage and the application of filters, are summarized in Table 1. The step-by-step selection process is visually illustrated in the Preferred Reporting Items for Systematic Reviews and Meta-Analyses (PRISMA) flow diagram, which highlights the progression from initial identification to final inclusion of eligible studies (Figure 1).

Data Extraction and Synthesis

Data extraction was conducted using a meticulously designed data extraction table, which was developed to systematically capture and organize relevant information from each included study (Table 2) [11-14,16,17,25,28-30]. The table was structured to encompass key elements essential for a comprehensive analysis of the impact of urgent care clinics (UCCs) on ED operations and patient outcomes. Specifically, the extracted data included the authors’ names, publication year, study objectives, study settings (e.g., geographic regions such as the United States or Canada), and sample characteristics, including patient demographics, volumes, and sampling methods.

**Table 2:** Summary of the results of the included studies. EMR: electronic medical record; MRD: Medical Record Department; LOS: length of stay

S. no.	1	2	3	4	5	6	7	8	9	10
Studies	Ho et al. [25]	Barzin et al. [29]	Hong et al. [11]	Allen et al. [28]	Park et al. [17]	Rothberg et al. [12]	Alhallak et al. [30]	Galloway et al. [14]	Bessert et al. [13]	D’Avella et al. [16]
Year	2017	2019	2019	2021	2022	2022	2021	2023	2023	2024
Place	USA	USA	USA	USA	USA	USA	USA	Canada	Germany	USA
Objective	To compare the usage and costs of freestanding emergency departments (EDs) vs. hospital-based EDs.	To evaluate the effectiveness of urgent care centers (UCCs) in reducing emergency department (ED) visits.	To assess the relationship between urgent care centers and emergency department visits.	To determine how urgent care centers affect emergency department demand.	To compare clinical and financial outcomes of urgent ophthalmic care at a triage eye clinic vs. ED referrals, evaluating visit duration, costs, and overall cost-effectiveness.	To assess the impact of the Oncology-specific urgent care center (OECC) on acute care utilization, particularly emergency department (ED) visits and hospital admissions among cancer patients.	Analyze the impact of Student-Run Free Clinics (SRFCs), specifically the Health and Wellness Center (HWC), on reducing non-urgent ED visits among uninsured patients.	To evaluate the impact of an urgent cancer care clinic on emergency department (ED) visits and overall health system performance.	To study the synergism of an urgent care walk-in clinic with an emergency department.	To compare emergency department (ED) utilization before and after the institution of a direct referral unit for oncology patients.
Settings	Freestanding EDs and hospital EDs UCCs.	Various urgent care centers in the United States.	Comprehensive cancer care facility.	Urgent care centers in the United States, using a novel dataset.	Tiage eye clinic.	Oncology-specific urgent care center (OECC) and EDs.	A student-run free clinic.	Urgent cancer care clinic within CancerCare Manitoba and related health institutions.	Urgent care walk-in clinic and emergency department.	Oncologic urgent care center. Single academic institution.
Sample	Data on freestanding EDs and hospital EDs utilization.	Average of 115 new patients per quarter established care after visiting the UCC.	33,316 adults aged 18 years or older diagnosed with incident cancer between July 2008 and December 2016. Excluded leukemia and non-melanoma skin cancer cases.	The study used state ED databases from AZ, FL, NE, NJ, NY, and RI, comparing ZIP codes with and without urgent care centers across various ED visit characteristics.	Initial total visits reviewed: 3,904 same-day visits. Exclusions due to incomplete records: 422 (including 204 "no-shows"). Final sample size for analysis: 3,482 initial presenting visits.	Pre-OECC period: 2,095 patients. Post-OECC period: 2,188 patients.	Included patient intake forms from 2024 (both return and new patient forms). Specific number of forms analyzed was not stated.	Cancer patients attending the urgent cancer care clinic.	Patients attending the urgent care walk-in clinic and emergency department.	Oncology patients on active treatment.
Sampling method	Retrospective comprehensive or convenience sampling (from records).	Retrospective comprehensive or census sampling (from EMR).	Non-probability sampling interrupted time series analysis.	Difference-in-differences analysis is employed to compare ED visit rates when urgent care centers are open vs. closed, within ZIP codes with and without centers.	Three-year retrospective chart review; consecutive sampling.	Retrospective, non-random sampling. MRD records.	Retrospective study.	Retrospective chart review of patients presenting with asthma exacerbations over a one-year period.	Prospective convenience sampling.	Retrospective observational study.
Strategy	Comparison of usage and cost data. Secondary analysis of insurance claims data to compare utilization patterns and costs across freestanding EDs, hospital-based EDs, and urgent care centers.	Employed difference-in-differences analysis to assess how UCCs affected ED demand trends over time.	Extended UCC hours to coincide with peak ED demand periods, optimizing patient accessibility and reducing ED visits.	Examination of how ED demand changes in the presence or absence of urgent care centers.	Patients were divided into two groups based on the site of initial presentation: patients presenting directly to the clinic ("TRIAGE"). Patients referred by the ED to the clinic ("ED + TRIAGE").	ED visit and hospitalization rates/100 patients pre- and post-OECC implementation.	Multivariable logistic regression to identify factors influencing the likelihood of patients seeking urgent care at the HWC instead of the ED.	Observational study with data analysis.	Pre-post comparative study.	Collaborated with healthcare partners to expand lab and radiology services, enhancing diagnostic capabilities and patient care efficiency.
Patient volume	There was a notable increase in visits to freestanding EDs (236%) and urgent care centers (24%) from 2012 to 2015, contrasting with a modest increase in hospital-based ED visits (10%).	Patient volumes stabilized around 900 patients/month. Average of 115 new patients per quarter established care after visiting the UCC. 122 million annual patient visits to UCCs.	4,846 patients had at least one ED visit within 180 days after cancer diagnosis during the post-UCC period. Only 589 patients (12.2%) completed 861 UCC visits during the same period.	Urgent care centers reduce total ED visits by 17.2% in ZIP codes where they are present. Reductions are significant for non-urgent visits (27%) and some emergent cases (e.g., avoidable emergent visits decrease by 3.6 visits).	Total analyzed visits: 3,482. Visits directly to the triage clinic: 2,538 (72.9%). Visits referred by the ED: 944 (27.1%).	Pre-OECC: 466 patients had 682 ED visits (32.6/100 patients). Post-OECC: 447 patients had 618 ED visits (28.2/100 patients).	About 10% of the HWC patient population used the clinic for emergencies.	Retrospective review of 186 patients aged two to 18 years. The urgent care clinic aimed to manage non-emergent cases of asthma exacerbations, suggesting a potential reduction in unnecessary visits to the ED for such cases.	Total patients (pre and post period): 10,176 ED patients recorded, 5,528 met inclusion criteria. Consenting participants: 4,765 (86.2%) of eligible ED patients; 1,201 (73.1%) of WIC patients.	Cohort from 2014 to 2018: 5,657 patients, 13,114 visits; 7,377 direct referral unit, 5,737 ED visits. Avg. 137 direct unit patients/month (range: 83-192); avg. 1.01 ED, 1.30 direct unit visits/patient.
Wait times	Specific wait time data are not provided in the text, but the increase in utilization at urgent care centers and freestanding EDs suggests potential differences in wait times compared to hospital-based EDs.	Impacts were concentrated among EDs with average wait times of over an hour.	Study highlights that longer ED wait times may encourage patients to use UCCs instead.	Urgent care centers have a pronounced effect in ZIP codes where EDs have longer wait times (e.g., reducing visits by 76.3% in EDs with the longest wait times).	"TRIAGE" group average visit duration: 158.2±78.3 minutes. "ED + TRIAGE" group average visit duration: 450.2±232.7 minutes. Difference: "TRIAGE" group saved an average of 292.2 minutes compared to the "ED + TRIAGE" group (p<0.001).	Not specifically mentioned.	Not explicitly mentioned.	By diverting non-emergent cases to the urgent care clinic, there is a potential to reduce wait times in the ED for more critical cases.	ED wait times: not specifically detailed, but the study notes a reduction in the percentage of low-urgency patients in the ED from 21.4% to 9.0% post-WIC opening. WIC wait times: mean length of stay in the WIC was 90.7±64.1 minutes.	Not specified.
Length of stay	Not specified.	Mentioned that fewer than 3% of visits resulted in direct hospital admission or transfer to ED.	Not specified.	Not specified.	Not specified.	OECC visits typically took more than 3 hours; additional delays were due to required COVID-19 tests and inpatient bed availability.	Not explicitly mentioned.	Not specified.	ED length of stay unchanged overall; self-referrals reduced by 17.6 mins, trauma surgery by 18.3 mins, dermatology increased by 35.1 mins. WIC stays 50% shorter than ED (91 vs. 172 mins).	LOS for admitted patients was similar between ED and direct referral unit post-adjustment: 5.5 days (95% CI: 5.1-5.9) for ED, 5.6 days (95% CI: 5.2-5.9) for direct referral unit (p=0.84).
Return to ED	Not specified.	Not specified.	After UCC visits, 9.3% resulted in an ED visit within 24 hours, and 7.5% of ED visits were followed by hospitalization.	The study suggests that urgent care centers may decrease return visits to the ED, especially for non-urgent cases, though specific return rates were not detailed.	Only 42 patients (1.2%) required an emergent workup in the ED after initial presentation at the triage clinic.	No specific data on return rates to the ED were provided.	Only 6% of emergency patients at HWC were referred to the ED.	Analysis of ED visit rates during urgent cancer care clinic hours suggests potential reduction in ED returns for non-emergent issues with specialized clinic care, though specific return rates were not detailed.	WIC to ED referrals: 16.4% of WIC patients were referred to the ED. Of the patients initially referred from the ED to the WIC, 17.4% were sent back to the ED.	Patients from the direct referral unit had higher subsequent ED or clinic visits within 30 days (p<0.004). Adjusted data revealed 27% of ED patients were admitted within 30 days vs. 22% from the direct referral unit (p<0.001).
Patient satisfaction	Not specified.	Not specified.	Direct measures of patient satisfaction with UCC visits or ED care were not reported.	Reduction in wait times and enhanced care access at urgent care centers may suggest improved patient satisfaction.	Reduced wait times and lower costs often correlate with higher patient satisfaction, despite lacking direct measures.	Positive feedback from patient comments in Press Ganey surveys, praising the OECC for its calming environment and provider expertise.	Not explicitly mentioned.	Not addressed.	Not explicitly mentioned in the provided content.	Not addressed directly.
Health outcomes	Not specified.	UCCs decrease non-emergent ED visits, with 33% of encounters potentially preventing ED visits. They address preventive or chronic disease needs in 25% of patients seen.	UCCs reduce ED visits across all four acuity levels, including non-urgent visits.	Urgent care centers are associated with a reduction in ED visits across all acuity levels, potentially mitigating overcrowding and its adverse outcomes, such as increased mortality and medication errors.	Low-acuity patients preferred the triage clinic over the ED (p<0.05). High-acuity cases, such as ocular trauma, mostly initiated care in the ED (59.9%; p<0.05). Most ophthalmic treatments were outpatient-based, with only 1.2% requiring emergent ED evaluation.	Post-OECC, ED visits decreased significantly by 4.6 per 100 patients. No notable change in overall or ED-triggered inpatient admissions. Increased early evening ED visits were observed post-OECC.	Not explicitly mentioned.	Analysis included the impact on ED visit rates by cancer type and severity.	Opening the WIC reduced low-urgency ED patients from 21.4% to 9.0%. Inpatient admissions rose from 21.9% to 28.3% post-WIC opening. Premature treatment discontinuation in the ED decreased from 6.4% to 3.6%.	Direct referral unit visits significantly increased chemotherapy/immunotherapy within 30 days (p<0.001). Common direct unit diagnoses included dehydration, nausea, and abdominal pain, while ED visits focused on symptoms, urinary issues, and respiratory concerns like fever and pneumonia.
Cost-effectiveness	Freestanding EDs impose high out-of-pocket costs on patients. Urgent care centers offer significantly lower costs than freestanding and hospital-based EDs, suggesting they are more cost-effective for non-emergent conditions.	Urgent care centers (UCCs) provide cost-effective care, saving $568,000 to $1,136,000 by averting ED visits. They achieved profitability within 18 months with financial backing, highlighting challenges without such support.	Estimated 488 fewer ED visits during the post-UCC period, suggesting potential cost savings associated with reduced hospital resource utilization. Highlighted the potential for cost-effectiveness in managing acute care needs of cancer patients.	Urgent care centers save an estimated $3.3 billion annually by reducing ED visits for non-urgent conditions, highlighting their cost-saving advantage over ED treatment for such cases.	The "TRIAGE" group incurred significantly lower charges and costs compared to the "ED + TRIAGE" group, with average savings of $3,847.50 and $578.40, respectively. ED visits showed higher Net Patient Service Revenue (NPSR) and contribution margin (CM), but under managed care and government plans, they resulted in financial loss.	OECC operating costs will be covered by enhanced oncology services payments during the OCM demonstration period. Potential for financial solvency through remittances across various payors and maximizing relative value units per provider-day.	Estimated cost savings of $39,515.80 in 2019 due to reduced ED visits if HWC was not available. Discussion on how SRFCs prevent EDs from incurring unnecessary costs.	Not directly addressed, but potential health system benefits discussed.	The study highlights walk-in clinics (WICs) as efficient, with shorter stays and adequate diagnostics for over 80% of ED referrals. It suggests that statutory health insurance-accredited urgent care could better suit about 30% of outpatient ED cases in Germany, reducing ED visits for outpatient care.	Direct referral unit visits showed significant cost savings compared to ED visits, with unadjusted charges averaging $2,221 vs. $10,261 for ED visits (p<0.001); adjusted costs were highlighted in savings for direct referral units.

Additionally, methodological details were carefully recorded, including the study design (e.g., retrospective chart reviews, prospective observational studies), analytical approaches, and any specific sampling techniques employed. A strong focus was placed on extracting key findings relevant to the review objectives, particularly metrics related to ED utilization (e.g., changes in patient volumes, wait times, and length of stay), patient outcomes (e.g., satisfaction, health improvements), and financial considerations (e.g., cost-effectiveness).

The data extraction table was iteratively refined to ensure consistency, clarity, and accuracy in data presentation across all included studies. Columns in the table were specifically designed to align with the review’s objectives, capturing vital information such as study identifiers (e.g., study number, author names, and publication year), study goals, sample descriptions, detailed methodologies, and the extracted outcomes. This structured format facilitated direct comparison of findings across studies and allowed for the identification of common themes, trends, and gaps in the evidence.

The analysis of the 10 included studies provides a detailed perspective on the role and impact of urgent care clinics (UCCs) within contemporary healthcare systems. The studies span diverse geographic locations and healthcare settings, with a primary focus on key parameters such as patient volume, wait times, length of stay, return visits to the ED, patient satisfaction, and health outcomes. Table 3 summarizes the characteristics and themes of the included studies.

**Table 3:** Summary of characteristics of the included studies.

Characteristics	Number of included studies (%)
Total	10 (100%)
Country	
United States	8 (80%)
Canada	1 (10%)
Germany	1 (10%)
Study type	
Retrospective	9 (90%)
Prospective	1 (10%)
Study design	
Comprehensive sampling	3 (30%)
Interrupted time series analysis	1 (10%)
Consecutive sampling	1 (10%)
Chart review	1 (10%)
Pre-post comparative study, convenience sampling	2 (20%)
Observational study	2 (20%)
Setting	
ED	1 (10%)
ED and urgent care centre or out-of-hours	3 (30%)
Student-run free clinic	1 (10%)
Urgent care center	3 (30%)
Direct referral unit	1 (10%)
Hospital/ED	1 (10%)
Key themes	
Cost-effectiveness	2 (20%)
Reduction in ED burden	3 (30%)
Improved health outcomes	6 (60%)
Reduced ED visits	2 (20%)
Patient satisfaction	2 (20%)
Efficiency of care	5 (50%)

Geographically, the studies were predominantly conducted in the United States (80%), with one study each from Canada and Germany, highlighting a regional concentration of UCC implementation and research in North America. Approximately 70% of the studies were situated in urgent or emergency care environments, such as EDs or urgent care centers, reflecting a strong focus on acute medical care. "Less frequently, settings such as student-run free clinics (a model that does not apply universally) and direct referral units were also represented, providing insight into the broader adaptability of UCCs in specific healthcare contexts."

In terms of study design, the majority (90%) were retrospective, relying on historical data to evaluate UCC impact, while only one study (10%) utilized a prospective design, indicating limited emphasis on future-oriented research methodologies within the dataset. Methodological diversity was evident, with designs including comprehensive sampling (30%), interrupted time series analysis, consecutive sampling, chart reviews, observational studies, and pre-post comparative studies. This range of approaches underscores the varied strategies employed to evaluate UCC performance and its implications for healthcare delivery.

Key themes identified across the studies included improved health outcomes (60%), efficiency of care (50%), reduction in ED burden (30%), cost-effectiveness (20%), and patient satisfaction (20%). These findings highlight the multifaceted role of UCCs in optimizing healthcare processes, reducing ED congestion, and enhancing patient care experiences.

Patient Volume

The included studies consistently demonstrated the significant impact of UCCs on patient volumes in EDs and related healthcare settings. Ho et al. reported a striking increase in visits to freestanding EDs (236%) and UCCs (24%) between 2012 and 2015, compared to a modest 10% increase in hospital-based ED visits [25]. Allen et al. observed that UCCs stabilized around 900 visits per month and reduced overall ED visits by 17.2% in ZIP codes where they were established, with a notable 27% reduction in non-urgent visits [28]. Similarly, Hong et al. highlighted that a significant proportion of cancer patients opted for UCC visits over ED visits after UCCs were introduced, though only 12.2% of these patients used UCC services [11]. Allen et al. reported a 17.2% reduction in total ED visits attributable to UCCs, particularly among non-urgent cases [28]. Alhallak et al. found that approximately 10% of uninsured patients in a student-run free clinic used the service for emergencies, reducing ED visits for non-urgent issues in this population [30].

Wait Times

UCCs were shown to play a significant role in reducing wait times across various healthcare settings. Barzin et al. demonstrated that UCCs led to a 76.3% reduction in ED visits in areas with longer ED wait times [29]. Park et al. compared visit durations between patients presenting directly to a triage clinic vs. those referred from the ED, finding significantly shorter visit durations for the former group (158.2 min vs. 450.2 min) [17]. Bessert et al. reported a reduction in low-urgency ED visits from 21.4% to 9.0% after a walk-in clinic (WIC) was established, indirectly improving ED wait times by diverting non-urgent cases to UCCs [13]. These findings underscore the ability of UCCs to alleviate ED congestion by providing an efficient alternative for non-emergent cases.

Length of Stay

The efficiency of UCCs in managing patient throughput compared to traditional ED settings was evident in the reported length of stay (LOS) outcomes. Barzin et al. noted that fewer than 3% of UCC visits resulted in direct hospital admissions, indicating shorter stays compared to ED visits [29]. Alhallak et al. reported an average LOS of 90.7 minutes in UCCs, significantly shorter than the average LOS of 172 minutes in EDs [30]. These results highlight the potential of UCCs to expedite care delivery for non-emergent cases, reducing the strain on ED resources and enhancing overall healthcare efficiency.

Return to ED

The impact of UCCs on return visits to the ED varied across studies. Hong et al. found that 9.3% of patients visiting UCCs returned to the ED within 24 hours, suggesting a subset of patients requiring further evaluation [11]. Park et al. reported that only 1.2% of patients seen at a triage clinic required emergent workups in the ED, demonstrating the effectiveness of UCCs in managing initial presentations [17]. Galloway et al. observed that 16.4% of UCC patients were referred to the ED, emphasizing UCCs’ role in appropriate triaging and directing patients to higher-level care when necessary [14]. D'Avella et al. reported higher subsequent ED or clinic visits within 30 days among patients from direct referral units compared to those initially presenting to the ED, reflecting ongoing challenges in ensuring care continuity [16].

Patient Satisfaction

Although direct measures of patient satisfaction were not reported in all included studies, several studies provided indirect evidence of improved patient experiences. Ho et al. and Barzin et al. suggested enhanced satisfaction due to reduced wait times and improved accessibility [25,29]. Rothberg et al. reported positive patient feedback in an oncology-specific UCC, highlighting satisfaction with specialized care and the clinical environment [12]. Bessert et al. attributed reduced wait times and focused care at UCCs to potentially higher satisfaction, though explicit satisfaction measures were not included [13].

Health Outcomes

The studies collectively illustrated the positive impact of UCCs on various health outcomes. Barzin et al. reported that UCCs prevented non-emergent ED visits in 33% of cases and addressed preventive or chronic disease needs in 25% of patients [29]. Allen et al. highlighted reductions in ER visits across all acuity levels, suggesting that UCCs help mitigate ER overcrowding and its associated adverse outcomes [28]. Park et al. noted that UCCs were preferred by low-acuity patients, supporting their role in managing appropriate care levels [17]. Bessert et al. emphasized that UCCs reduced low-urgency ED visits and facilitated a more efficient allocation of healthcare resources, ultimately contributing to improved patient outcomes [13].

Quality Assessment

The quality assessment for the included studies was assessed using the Newcastle-Ottawa Scale (NOS). Among the 10 studies, six were classified as high quality (7-9 points) and four had a moderate risk of bias (4-6 points).

High-quality studies had well-defined selection criteria, appropriately controlled for confounders, and used validated outcome measures. Studies with moderate quality showed some limitations in participant selection or adjustment for confounders but maintained acceptable outcome assessment reliability. The lowest score was related to Alhallak et al.'s study [30]. This low score was due to unclear participant selection criteria, inadequate confounder control, and reliance on self-reported outcome measures with incomplete follow-up data. This introduces potential selection, comparability, and outcome biases, reducing the study’s reliability. A summary of the quality assessment is presented in Table 4.

**Table 4:** Quality assessment of the included studies.

Study	Selection (4)	Comparability (2)	Outcome (3)	Total score (max 9)	Quality of study
Ho et al. (2017) [25]	3	2	2	7	🟢 High
Barzin et al. (2020) [29]	4	2	3	9	🟢 High
Hong et al. (2019) [11]	3	1	2	6	🟡 Moderate
Allen et al. (2021) [28]	3	2	2	7	🟢 High
Park et al. (2022) [17]	4	2	3	9	🟢 High
Rothberg et al. (2022) [12]	3	1	2	6	🟡 Moderate
Alhallak et al. (2021) [30]	2	1	1	4	🟡 Moderate
Galloway et al. (2023) [14]	3	2	2	7	🟢 High
Bessert et al. (2023) [13]	3	1	2	6	🟡 Moderate
D'Avella et al. (2024) [16]	4	2	3	9	🟢 High

Discussion

The growing prevalence of urgent care clinics (UCCs) represents a significant shift in healthcare delivery, particularly in managing non-life-threatening conditions and reducing ED congestion. UCCs offer walk-in services with extended hours, providing timely, cost-effective care for patients who might otherwise seek care at EDs [31]. Their role in alleviating ED overcrowding has been widely explored, with evidence indicating both successes and challenges in achieving this goal.

Patients often seek care in EDs for non-urgent conditions due to perceived urgency or confusion regarding healthcare navigation, which unnecessarily burdens ED resources. According to Thijssen et al., patients frequently default to ED visits because they are unaware of alternative care settings, such as UCCs, that could meet their healthcare needs more efficiently [31]. Educational interventions and clearer triage guidelines are essential to improve patient healthcare navigation and ensure the appropriate use of healthcare services [32].

Several studies demonstrate the potential of UCCs to reduce non-urgent ED visits [33,30]. For example, Ho et al. reported a significant increase in visits to UCCs, suggesting a growing preference for these alternative care settings, particularly when they offer shorter wait times and convenient access [25]. In contrast, Barzin et al. observed that UCCs experienced stabilized patient volumes over time, indicating their role in providing sustainable diversionary care for non-urgent cases [29].

Regional Differences

Comparing GP cooperatives, walk-in clinics, and UCCs highlights different approaches to managing non-urgent healthcare needs. GP cooperatives focus on localized primary care services aimed at reducing ED attendances, particularly by providing personalized care and shorter wait times [18]. In contrast, UCCs are designed to handle a broader range of urgent conditions, offering more comprehensive services to reduce ED utilization [17]. The research shows that UCCs are more effective in reducing non-urgent ED visits and improving patient satisfaction through timely and efficient care [13,33].

However, regional variations in healthcare systems and population demographics influence the effectiveness of UCCs in reducing ED congestion. Allen et al. found that areas with UCCs experienced a reduction in ED visits, particularly for low-acuity cases, whereas Alhallak et al. suggested that vulnerable populations may require more targeted interventions to ensure equitable access to urgent care services [28,30,34]. These findings highlight the need for tailored approaches that account for local healthcare demands and population-specific needs.

Specialty urgent care clinics, such as oncology-specific UCCs and triage eye clinics, further demonstrate the value of targeted healthcare delivery models. Bessert et al. found that specialty clinics reduced ED referrals for specific conditions, improving patient outcomes and healthcare system efficiency [13]. These clinics focus on specialized care, reducing ED congestion while addressing unique patient needs [17].

Healthcare systems worldwide have different structural models for urgent care services. In the United Kingdom, urgent treatment centres (UTCs) operate under the following two primary models: colocated UTCs that function alongside emergency departments, and standalone UTCs situated within communities. Both models are designed to reduce ED attendance, though their effectiveness may vary based on local demographics and healthcare needs. Understanding these structural variations and system-specific factors is crucial for evaluating and implementing effective urgent care services across different healthcare systems.

Cost-Effectiveness

Despite these benefits, freestanding EDs - which offer emergency care in non-hospital settings - present both opportunities and challenges. Ho et al. noted a rise in visits to freestanding EDs, driven by patient preferences for convenience and shorter wait times [25]. However, these facilities are associated with higher costs, raising concerns about their financial sustainability compared to UCCs [25].

In summary, UCCs play a crucial role in reducing ED congestion and improving healthcare efficiency. Their success depends on patient education, system integration, and appropriate healthcare navigation mechanisms. Moving forward, policy efforts should focus on strengthening the integration of UCCs within broader healthcare systems to enhance healthcare delivery and patient outcomes [13,28,33].

Limitations of UCCs

This systematic review presents several limitations that should be acknowledged. The included studies exhibit substantial variability in their methodologies, sample sizes, different groups of patients, and geographic focus, introducing heterogeneity that may affect the consistency of the findings and limit their applicability across different healthcare systems.

Moreover, the review predominantly concentrates on quantitative outcomes, such as emergency room visit rates, patient satisfaction, and cost savings, largely derived from retrospective analyses of administrative data. This reliance on retrospective, quantitative data may contribute to inherent biases, including publication bias, as studies with positive outcomes related to alternative care models are more likely to be published.

A further limitation is the limited geographic and linguistic scope of the included studies. The majority of studies originate from North America and other English-speaking regions, and only research published in the English language was considered. This focus may not fully capture the complexities of healthcare systems in non-English-speaking countries or contexts where UCCs are structurally integrated within emergency departments. Consequently, relevant research from other regions may have been excluded, and structural and operational differences that significantly influence care delivery and system performance may have been overlooked, introducing both geographical and language bias.

Future research should seek to address these limitations by incorporating a more diverse range of study designs, including qualitative analyses, and expanding the scope to include international and multilingual sources. Such efforts would enhance the robustness, depth, and global representativeness of the conclusions drawn.

## Conclusions

The findings from this review highlight the critical role of urgent care clinics (UCCs) in alleviating emergency department congestion and improving healthcare efficiency. By offering accessible, cost-effective care for non-life-threatening conditions, UCCs reduce non-urgent ED visits, improve patient satisfaction, and optimize healthcare resource allocation.

Freestanding EDs and specialty clinics also contribute to diversifying healthcare delivery models, although their cost-effectiveness requires careful evaluation. Moving forward, healthcare systems must prioritize the integration of UCCs and other alternative care settings to address ongoing challenges related to ED overcrowding and increasing healthcare demands. Future research should explore long-term patient outcomes, health system sustainability, and regional variations to optimize the impact of UCCs on ED utilization and patient care experiences.
